# Using ionic liquids in whole-cell biocatalysis for the nucleoside acylation

**DOI:** 10.1186/s12934-014-0143-y

**Published:** 2014-10-02

**Authors:** Meiyan Yang, Hui Wu, Yan Lian, Xiaofeng Li, Yao Ren, Furao Lai, Guanglei Zhao

**Affiliations:** State Key lab of Pulp & Paper making Engineering, South China University of Technology, Guangzhou, 510641 China; College of Light Industry and Food Sciences, South China University of Technology, Guangzhou, 510641 China

**Keywords:** Biocatalysis, Whole-cells, Ionic liquid, Nucleoside ester, Regioselectivity

## Abstract

**Background:**

The use of biocatalysts has become an increasingly attractive alternative to traditional chemical methods, due to the high selectivity, mild reaction conditions and environmentally-friendly processes in nonaqueous catalysis of nucleosids. However, the extensive use of organic solvents may generally suffer from sever drawbacks such as volatileness and toxicity to the environment and lower activity of the biocatalyst. Recently, ionic liquids are considered promising solvents for nonaqueous biocatalysis of polyhydroxyl compounds as ILs are environmental-friendly.

**Results:**

In this research, we developed new IL-containing reaction systems for synthesis of long chain nucleoside ester catalyzed by *Pseudomonas fluorescens* whole-cells. Various ILs exerted significant but different effects on the bio-reaction. And their effects were closely related with both the anions and cations of the ILs. Use of 10% [BMI][PF_6_]/THF gave high reaction efficiency of arabinocytosine laurate synthesis, in which the initial rate, product yield and 5′-regioselectivity reached 2.34 mmol/L·h, 81.1% and >99%, respectively. Furthermore, SEM analysis revealed that ILs can alter the cell surface morphology, improve the permeability of cell envelopes and thus facilitate the mass transfer of substrates to the active sites of cell-bound enzymes.

**Conclusion:**

Our research demonstrated the potential of ILs as promising reaction medium for achieving highly efficient and regioselective whole-cell catalysis.

## Background

The use of biocatalysts has become an increasingly attractive alternative to traditional chemical methods, due to the high selectivity, mild reaction conditions and environmentally-friendly processes [1,2]. Biocatalytic processes can be performed by using isolated enzymes or whole-cells. Whole-cell biocatalysts utilize the cell-bound enzymes as well as intracellular enzymes which are biosynthesized during cell growth. The cooperation of a serial of different kinds of cell-bound enzymes and/or intracellular enzymes forms an efficient whole-cell catalyst. Most isolated enzymes are extracellular ones which are fermented by the microbes and prepared via separation and purification from the fermentation broth [3]. In the past decades, there are tremendous progresses in the use of enzymes. The major drawbacks of enzyme catalysts are their much higher costs, since they need time-consuming and multiple purification processes. In some cases, removal of an enzyme from its natural cell environment may lead to partial or even completely loss of the enzyme activity [4]. The commonly-used immobilization technologies endow them with recycling properties, but made them even more expensive [5]. In addition, the variety of commercial enzymes was limitedly available for a specific reaction. The researchers have to screen microbe strains from natural environment and separate enzymes from the cells. Contrary to the limited varieties of enzymes, microorganisms are widely distributed in nature. Direct use of microbial whole-cells as biocatalysts instead of isolated enzymes is a potential way to reduce the cost of industrial process, since they could avoid the tedious preparation procedures of the enzymes and maintain the enzyme activity by protecting the cells. Several processes have benefited from whole-cell biocatalysis, including production of biodiesel [6] and other high-value compounds [7]. However, compared with isolated enzymes, whole-cells gained so less attention in both researches and applications [2].

Regioselective acylation of polyhydroxyl compounds is a well-known difficult task and great challenge to organic chemists [8]. Nucleoside analogues, a typical kind of those compounds, are also highly valuable in pharmaceutical [7-10]. Enzymatic modification strategy has been proposed and proven to be effective for new nucleoside drug/prodrug discovery and development. A lot of nucleosides esters were thus produced with higher antitumer activities than their original forms [9,10], among which the long-chain fatty acid esters of nucleosides showed higher bioactivity than those with short chains [11]. However, the above-mentioned high cost of enzymatic approaches is a significant limitation hampering their industrial applications [2,12]. Thus considerable research is needed to develop new kinds of catalysts. To our knowledge, no reports have been made on the synthesis of long-chain nucleoside esters by employing whole-cells biocatalysts.

Besides the catalysts, the choice of reaction media is also considered as an important issue [1,13-15]. The extensive use of organic solvents may generally suffer from several drawbacks such as volatileness and toxicity to the environment. The use of alternative reaction media has been receiving increasing attention which should circumvent the problems associated with many of the traditional volatile organic solvents [15]. Recently, ionic liquids (ILs), a class of organic salts with melting points below 100°C, have attracted a lot of attention as possible replacement for conventional molecular solvents for catalysis. They exhibit unique properties including nonvolatility, nonflammability, and excellent chemical and thermal stability. Over the past decade, the use of ILs in biocatalysis for organic synthesis has been extensively studied [12,16]. In many cases, the biocatalysts showed higher catalytic activity, selectivity and operational stabilities when employing ILs as reaction media. However, ILs have yet to be widely applied in industry, which are mainly related to their high price and less knowledge about their toxicity and biodegradability [15]. Investigation of the IL toxicity on the microbial cells with catalytic activities is of great significance since it can provide the toxicity data of ILs in the field of catalysis, thus achieving new advances in biocatalysis and promote the development of nontoxic, biodegradable ionic liquids [17].

**Scheme 1:**
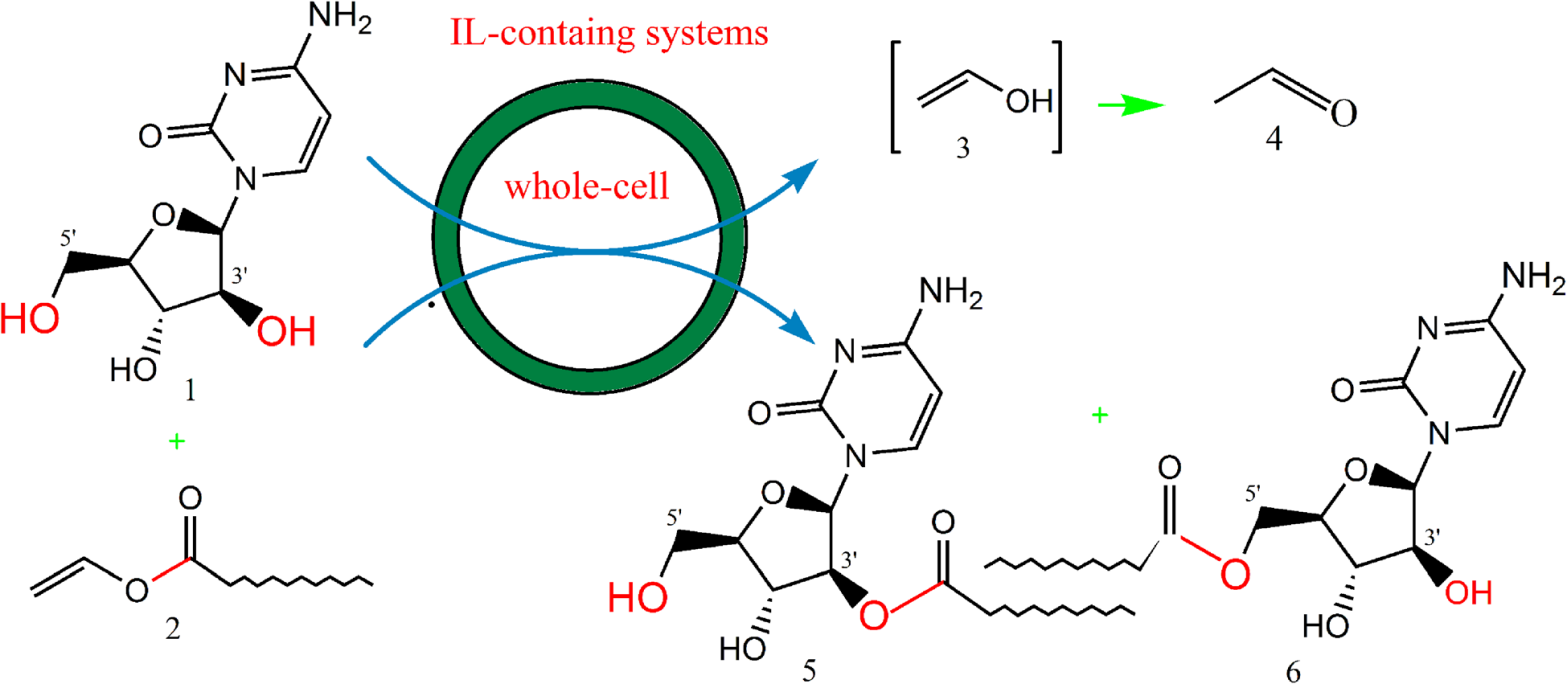
**Transesterification of ara-C with vinyl laurate catalyzed by whole-cell (1:Ara-C; 2:VL;3:Unstable enol; 4: Aldehyde; 5: 5′-O-lauryl ara-C; 6: 3′-O-lauryl ara-C).**

Encouraged by our recent achievements in whole-cell biotransformation, we tried, for the first time, the synthesis of long-chain nucleoside esters in ILs-containing systems. Synthesis of laurate ester of arabinocytosine (ara-C), a clinical antitumor drug, was employed as a model reaction (Scheme 1). The cells of *Pseudomonas fluorescens* GIM1.209 were used as the biocatalyst. The influence of ILs of different types on the whole-cell catalyzed reaction was investigated. And the effects of ionic liquids on the cell morphology of the microbes were evaluated in terms of the biomass and surface morphology of the cells.

## Result and discussion

### The influence of different ILs on the catalytic activity of the whole-cells

Previous reports demonstrated that certain ILs had attractive property in dissolving polar compounds [18,19]. And the properties of ILs largely depend on their cations and anions. To cope with the noticeable mass transfer problem caused by the relatively high viscosity of ILs, we tried to mix pure ILs with a typical organic solvent THF to form different IL-containing systems. As shown in Table 1 (Entries 1-4), *P. fluorescens* catalyzed acylation proceeded in four [BF_4_]^-^ based IL-containing systems. Although the addition of ILs obviously slowed down the reaction rates, the addition of IL [BMI][PF_6_] to THF greatly improved the yield of the whole-cell mediated reaction from 4.58% to 81.05%. It was very interesting since only 10% IL used gave such great promotion on the reactions. It was also found that the yield of the reaction increased evidently with elongating the carbon chain length of the cations of the ILs tested. Excepted for [EMI][BF_4_]-containing system, the initial rates of the reaction in other [BF_4_]^-^ based ILs system also showed a increasing tendency with increasing the carbon chain length of the cations. As reported previously, the hydrophilicity of the anions of [BF_4_]^-^ based ILs decreased with elongating the carbon chain length [20]. When the anion in the IL changed from [BF_4_]^-^ to [PF_6_]^-^, both the yield and initial rate of the reaction were markedly enhanced to the highest values, being of 81.05% and 2.34 mmol/L·h, respectively, much higher than those in [BF_4_]^-^ based ILs and traditional organic solvent tested (data not shown). No product was detected when the ILs containing halogen anions, namely [BMI][Br] or [BMI][Cl]. These results indicated that the IL with lower hydrophilicity might favor the whole-cell mediated acylation, since the hydrophilicity of the anions increases in the order of [halide ions]^-^ > [BF_4_]^-^ > [PF_6_]^-^ [20]. In the imidazolium cation-based ILs-containing systems, [BMI][TF_2_N] is more hydrophobic than [BMI][PF_6_] [21], the initial rate of the reaction in [BMI][TF_2_N] was higher than that in [BMI][PF_6_]-containing system (Table 1, Entry 9), showing that hydrophobicity of IL may be a key factor influencing the cell-bound lipases. Comparison of the reactions performed in the same cation [BMI]^+^-containing ILs indicated that the anion type of the ILs played a crucial role in whole-cell mediated acylation [12,16,22]. For example, the [PF_6_]^-^ , an enzyme-compatible anion, may had lower hydrogen bond basicity, thus minimizing interference with the internal hydrogen bonds of the enzyme.Although different IL-containing systems were adopted, the 5′-regioselectivity of the whole-cells remained as high as >99%. Considering both the reaction efficiency and regioselectivity, [BMI][PF_6_]-containing system was chosen for further investigation.

**Table 1:** **Acylation of ara-C with VL by** ***P. fluorescens*** **in IL-containing systems** ^**a**^

**Entry**	**Solvents** ^**b,c**^	**V**_**0**_ **(mmol/L · h)**	**Y(%)**	**5′-regioselectivity(%)**
1	THF	2.42	4.6	>99.0
2	[EMI][BF_4_]/THF	0.05	2.4	>99.0
3	[BMI][BF_4_]/THF	0.03	3.2	>99.0
4	[HMI][BF_4_]/THF	0.23	4.8	>99.0
5	[OMI][BF_4_]/THF	0.25	7.5	>99.0
6	[BMI][PF_6_]/THF	2.34	81.1	>99.0
7	[BMI][Br]/THF	0	0	0
8	[BMI][Cl]/THF	0	0	0
9	[BMI][TF_2_N]/THF	3.15	70.4	>99.0

^a^The reaction conditions: 50 mg/mL biomass, 20 mmol/L ara-C, 500 mmol/L VL, 40 μL water, 1 mL solvents, 40°C, 200 rpm and 144 h. ^b^IL content, 10% v/v; ^c^Pyridine (10%, v/v) was added to the reaction media as an assistant solubilizer for dissolving the substrate.

### Influence of ILs concentration on the reaction

**Figure 1:**
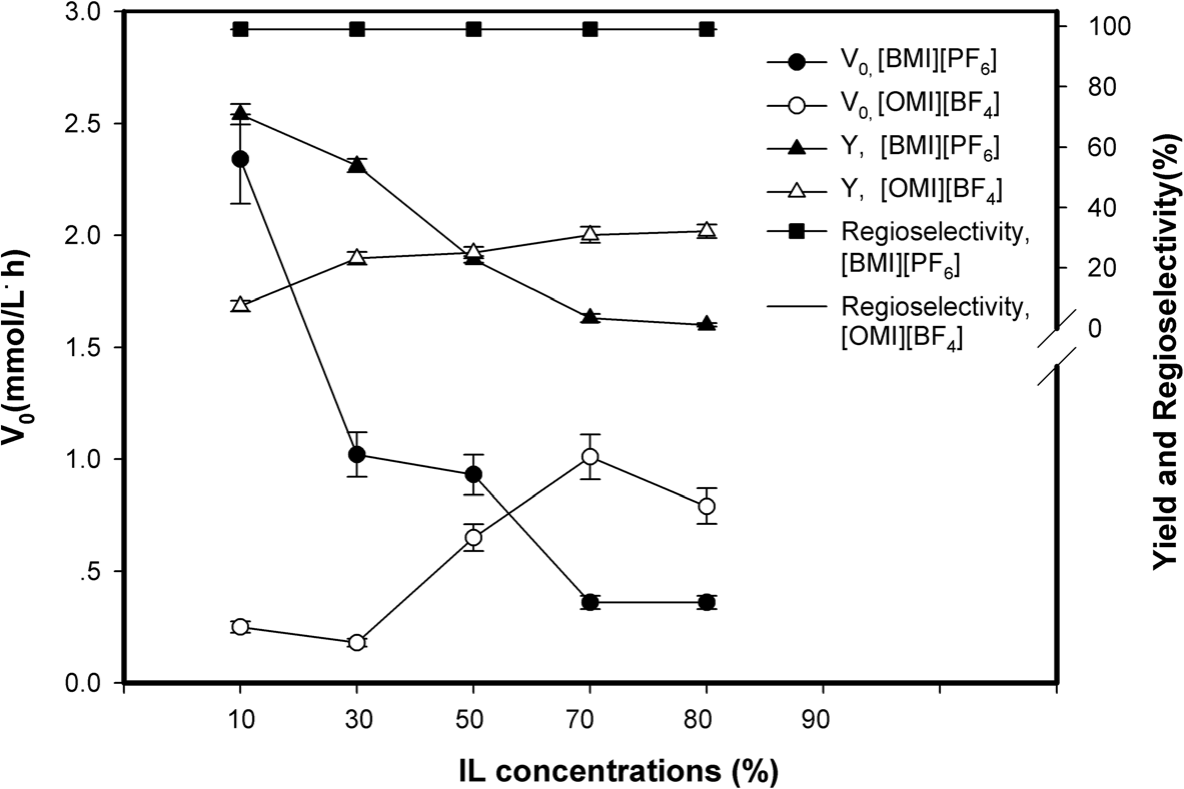
**Effects of IL contents on the initial rates, yields and 5′-regioselectivity of the transesterification of ara-C with VL by** ***P. fluorescens*** **in IL-containing systems (Reaction conditions: 50 mg/mL biomass, 20 mmol/L ara-C, 900 mmol/L VL, 40 μL water, 1 mL reaction media, 40°C, 200 rpm and 144 h).**

The effect of the amount of IL in the IL/THF system containing [BMI][PF_6_] was further investigated and compared with that of [OMI][BF_4_], as [OMI][BF_4_] showed to be the best one among the [BF_4_]^-^ based ILs system tested. As shown in Figure 1, in [OMI][BF_4_]-containing system, an increase in IL content up to 80% (v/v) brought about a clear increase in product yield of the reaction, both the initial reaction rate and product yield of the reaction declined significantly as the increase of [BMI][PF_6_] concentration when using IL-containing solvent as reaction medium. The possible reason for the increase phenomenon resulted by increasing IL content observed in [OMI][BF_4_]-containing system was that this IL might enhance cell membrane permeability by hydrolyzing certain components of the cell wall when the cells fully dispersed and contacted with them, and thus accelerating the catalytic reaction [23]. Our experiments also showed that the dispersibility of lyophilized *P. fluorescens* cells powder in [BMI][PF_6_] was lower than in [OMI][BF_4_]. More apparent agglomeration phenomenon of the lyophilized cell powder appeared in higher content of [BMI][PF_6_] system, which may reduce the catalytic efficiency of the cell powder and aggravate the mass transferring limitations of the substrates. Changes in the IL contents showed little effect on 5′-regioselectivity of the reaction (above 99%).

### Effect of organic cosolvents in IL-containing system

Inspired by the interesting phenomenon obtained by different IL-containing systems listed above, we tried the combination of IL (in terms of [BMI][PF_6_] or [OMI][BF_4_]) with different type of organic solvent for the reaction. Table 2 (Entries 1-5) showed the reactions performed in the systems containing [OMI][BF_4_] (at its optimized volume content, 80%) mixed with different types of organic solvents with increased hydrophobility (log*P* value). The changes in the types of organic co-solvents resulted in quite different initial rates, product yields and even regioselectivity of the whole-cell catalyzed reaction. For the cases that acetone or *t*-butanol was used as co-solvents, the 5′-regioselectivity of the reactions were decreased. In [BMI][PF_6_]-containing systems (Table 2, Entries 6-10), both the initial rate and the product yield declined as the log*P* value of the solvent-assistant increased except for that in [BMI][PF_6_]/acetone system, indicating that the hydrophobicity of the cosolvents may play an important role in mediating the whole-cell catalyzed acylation. Besides their effects on cell-bound enzymes, different organic solvent molecules of solvation shell may surround the substrates and thus can be regarded as loosely bound substituents to modify the chemical reactivity of the substrates [24]. The results also demonstrated that, the use of ILs for partially replacement of traditionally organic solvents not only made the whole process easily handled, but also improved the process performance.

**Table 2:** **Effect of different organic cosolvents on acylation of ara-C by** ***P. fluorescens*** **in IL-containing systems** ^**a**^

**Entry**	**IL-containing system**	**log** ***P*** **value** ^**e**^	**V**_**0**_ **(mmol/L·h)**	**Y(%)**	**3′-regioselectivity (%)**	**5′-regioselectivity (%)**
1	[OMI][BF_4_]/acetone^b,d^	-0.23	0.74	9.4	10.5	89.5
2	[OMI][BF_4_]/THF^b,d^	0.49	0.77	32.1	<1.0	>99.0
3	[OMI][BF_4_]/*t*-butanol^b,d^	0.80	1.06	13.5	8.25	91.8
4	[OMI][BF_4_]/Isopropyl ether^b,d^	1.90	0.80	12.9	<1.0	>99.0
5	[OMI][BF_4_]/hexane^b,d^	3.50	1.11	14.4	<1.0	>99.0
6	[BMI][PF_6_]/acetone^c,d^	-0.23	1.56	26.1	<1.0	>99.0
7	[BMI][PF_6_]/THF^c,d^	0.49	2.34	81.1	<1.0	>99.0
8	[BMI][PF_6_]/t-butanol^c,d^	0.80	1.98	18.8	<1.0	>99.0
9	[BMI][PF_6_]/Isopropyl ether^c,d^	1.90	0.64	9.0	<1.0	>99.0
10	[BMI][PF_6_]/hexane^c,d^	3.50	0.37	5.3	<1.0	>99.0

^a^The reactions conditions: 50 mg/mL biomass, 20 mmol/L ara-C, 900 mmol/L VL, 40 μL water, 1 mL reaction media, 40°C, 200 rpm and 144 h. ^b^[OMI][BF_4_] content, 80% (v/v); ^c^[BMI][PF_6_] content, 10% (v/v); ^d^10% Pyridine (v/v) was added as an assistant solubilizer for dissolving ara-C. ^e^log*P* value of the organic cosolvents, referred to Ref. (Trends in Biotechnology, 1995, 13(2):63-70).

### Stability of the whole-cells in different reaction system

**Figure 2:**
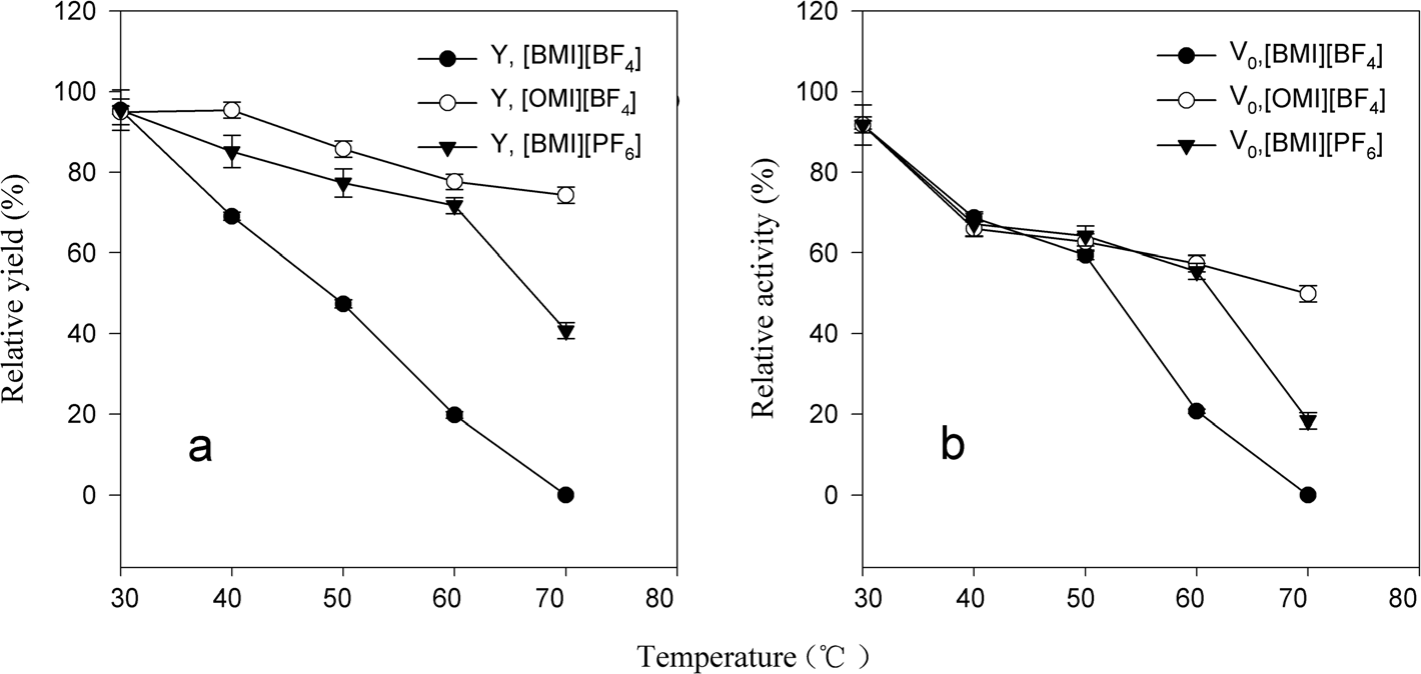
**The thermal-stability of** ***P. fluorescens*** **whole-cells in IL-containing systems.** [**(a)** Relative yield; **(b)** Relative activity)].

From both a practical and a theoretical viewpoint, it was of considerable interest to understand the stability of whole-cells in ILs since the commercial application of biocatalyst is often impeded by the short half-life. Figure 2 illustrates the deactivation profile of whole-cells in different media ([BMI][PF_6_]-THF, [OMI][BF_4_]-THF and [BMI][BF_4_]-THF). After incubation at 40°C for 12 h, similar relative catalytic activities of the whole-cells were found in the three different IL-containing systems, nearly 70% of the original catalytic activity. When the incubation temperature increased from 40 to 60°C, the residual activity of whole-cells in [BMI][PF_6_] and [OMI][BF_4_]-THF systems kept above 60%, while that in [BMI][BF_4_]-THF system sharply dropped down to ~20%. Higher incubation temperature than 60°C resulted in sharp decrease in residual activities of the cells in IL-THF systems. These results suggested that different kinds of ILs may have different effect on the stability of the cells. The different interactions of ILs with the active site of the cell-bound lipases should be speculated to explain these results [25]. The difference in electrostatic interactions between ILs and the cell-bounded protein may cause varied rigidity of the proteins, resulting in the thermal-stability variation of the biocatalysts [26]. In addition, the ILs may have different toxic effects on the cell morphology of the whole-cell biocatalyst, which may also account for observed variations in thermal-stabilities of the whole-cells.

### Effects of different IL-containing systems on surface morphology of the whole-cells

**Figure 3:**
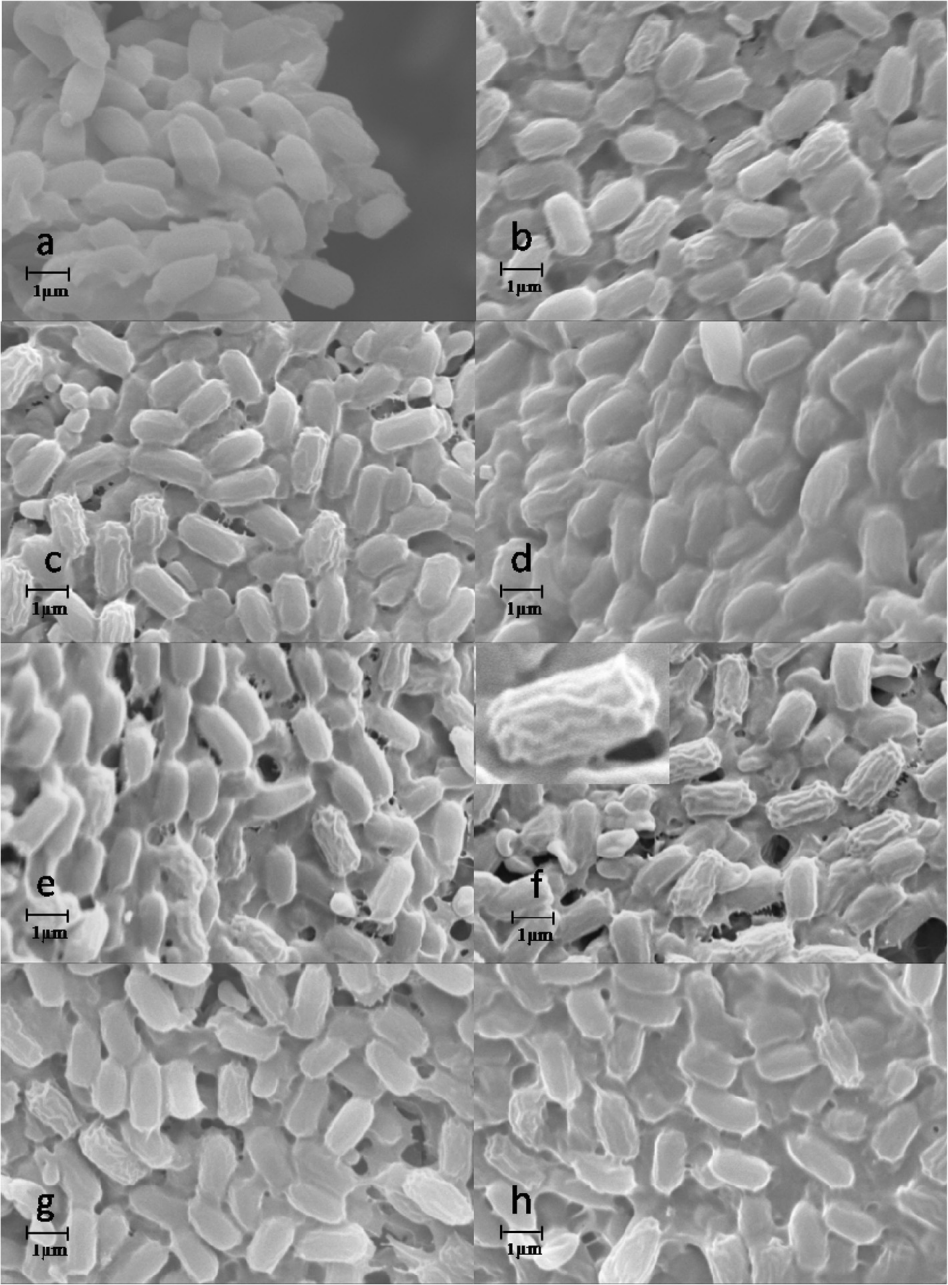
**SEM analysis of cell morphology of** ***P. fluorescens*** **incubated in different IL-containing non-aqueous media (a: PBS buffer, b: [EMI][BF**_**4**_ **]/THF, c: [BMI][BF**_**4**_ **]/THF, d: [HMI][BF**_**4**_ **]/THF, e: [OMI][BF**_**4**_ **]/THF, f: [BMI][PF**_**6**_ **]/THF, g: [BMI][Br]/THF, h: [BMI][Cl]/THF).**

For whole-cells mediated reactions, ILs might affect both the enzymes bounded on/in cells and the cell structures. However, the mechanisms of IL toxicity to microorganisms remain poorly understood [27,28]. To gain insight into the influence mechanism of ILs on the whole-cells catalyzed transesterification, equivalent dosages of the lyophilized cells were incubated in different IL-containing systems for SEM analysis. Figure 3 illustrated that,after incubated in PBS buffer solution (control) for 24 h, the cells showed the normal shapes of *P. fluorescens* with smooth surfaces (Figure 3a). When incubated with different ILs/THF systems (IL content 10%, v/v),evident wrinkling of the cells were found (Figure 3b-h). The quality of wrinkled cells varied with different ILs used. And the wrinkling phenomenon was more evident in [BMI][PF_6_]-containing system than in other systems. The use of halogen ions (Br^-^ or Cl^-^) based ILs led to less wrinkling of cell envelope, indicating that this kind of ILs had less influence on cell structures than the other ILs. Thus the observed loss in total catalytic activity of whole-cell biocatalyst in the transesterification performed in the halogen ions (Br^-^ or Cl^-^) based ILs may be mainly due to their inactivation effect on the enzymes located in the cells.

**Figure 4:**
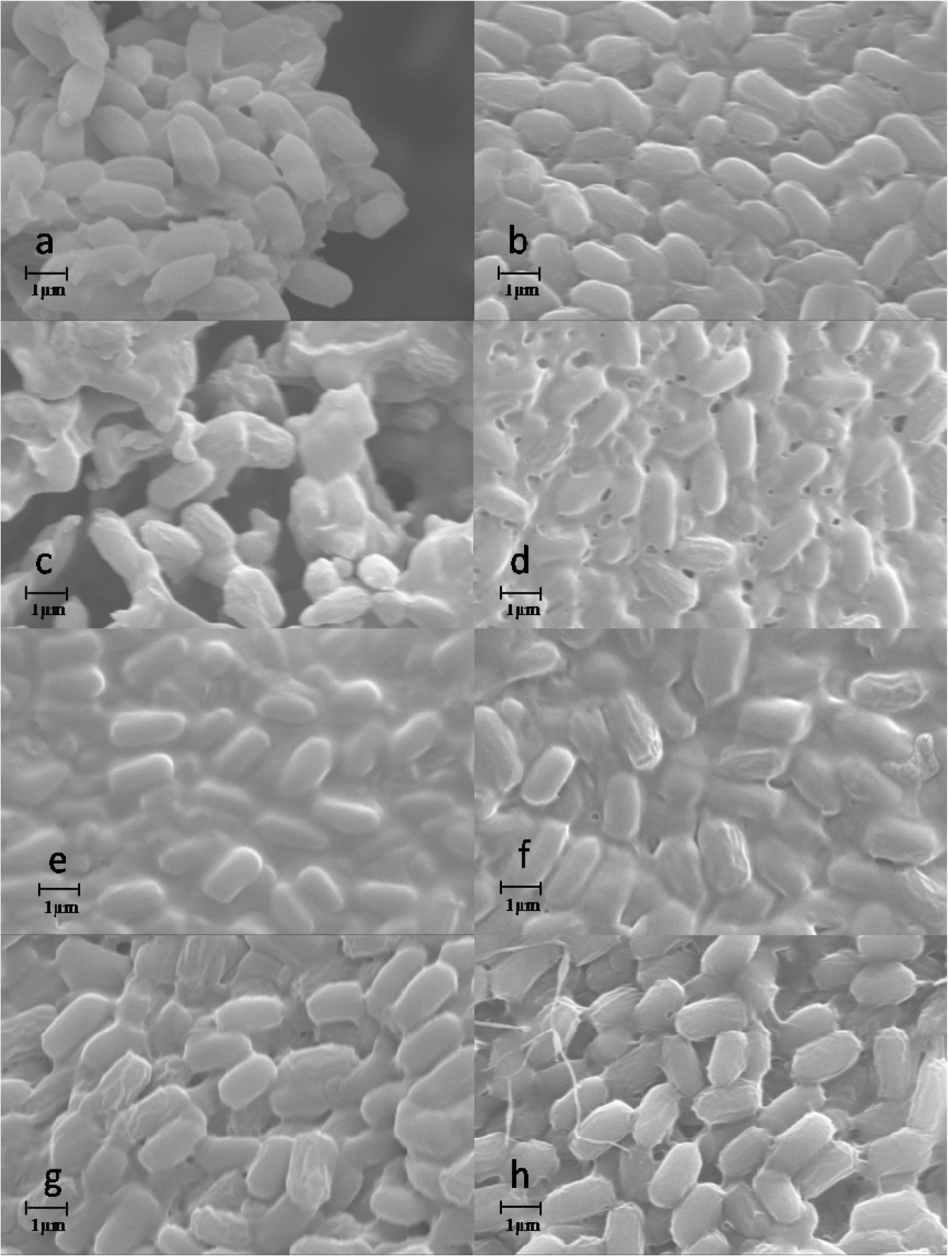
**SEM analysis of cell morphology of** ***P. fluorescens*** **incubated in different pure ILs (a: PBS buffer, b: [EMI][BF**_**4**_ **], c: [BMI][BF**_**4**_ **], d: [HMI][BF**_**4**_ **], e: [OMI][BF**_**4**_ **], f: [BMI][PF**_**6**_ **], g: [BMI][Br], h: [BMI][Cl]).**

To confirm that ILs in the solvent mixtures contributed to the wrinkling of the cells, we then incubated the cells with different pure ILs, and subsequently analyzed the cell surfaces by SEM. Figure 4 showed that for all the cells incubated in pure ILs used, wrinkling of the cell surface can also be observed, but their wrinkling levels in pure ILs was much lower than those in the corresponding IL/THF mixtures, suggesting a synergistic effect of the IL and organic solvents on cell surface.

The interesting wrinkling of the wall envelope of whole-cells treated with ILs clearly showed that the ILs tested had toxicity effects on the cells of *P. fluorescens P. fluorescens* is one of the typical strains of Gram-negative bacteria, which has thinner peptidoglycan layer and an additional outer membrane. Both the membranes mainly consist of a lipid bilayer in which a large number of enzymes and transport proteins may be embedded [6,29,30]. The hydrophobic ions (such as [PF_6_]) endow the ILs with lipophilicity (like ionic surface active agents), thus facilitating interactions with outer membrane and then the plasma membrane of the cells [31]. Thus the mechanism of observed IL toxicity to the bacterial cells may be via membrane accumulation, disruption and subsequent increase of permeability of both cell wall and plasma membrane [32,33]. And therefore, some cellular contents of the cells may be leaked into the solvents. From the point of view of whole-cell biocatalysis, partial disruption and increased permeability of the cell wall and plasma membrane may reduce the inner mass-transfer limitation of the substrates into the enzyme molecules bonded on cells, thus improve reaction efficiency. Bradford Protein Assay KitSuper showed detectable protein contents in IL-containing reaction mixtures after separation of the cells from the media, which confirmed our conclusion of IL influence on cell permeability deduced based on the SEM data.

### ILs toxicity to cell biomass

**Figure 5:**
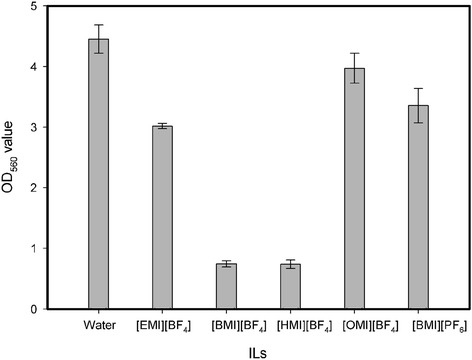
**OD**_**560**_ **values of the media of** ***P. fluorescens*** **cultivated by adding 5% (v/v) ILs.**

Figure 5 depicts the effects of different IL-containing systems on the biomass of the cells of *P. fluorescens*. Obvious decrease in cell concentration appeared when all the ILs were tested, indicating ILs may interrupt the reproduction of the *P. fluorescens* cells. No relations between the chain lengths of the [BF_4_]-based ILs with cell concentration was found. When cultured with [OMI][BF_4_] and [BMI][PF_6_], the cell growth of *P. fluorescens* showed comparable values to that of control group. This implies that the toxicity effects of the ILs on bacterial cells were closely related to their types. And, [OMI][BF_4_] and [BMI][PF_6_] may have relatively lower inhibition effects on cell growth than other ILs tested. Therefore, the effect of an IL as the nonaqueous reaction medium on mature cells may be different from that as culture additives on biomass of the cells.

**Figure 6:**
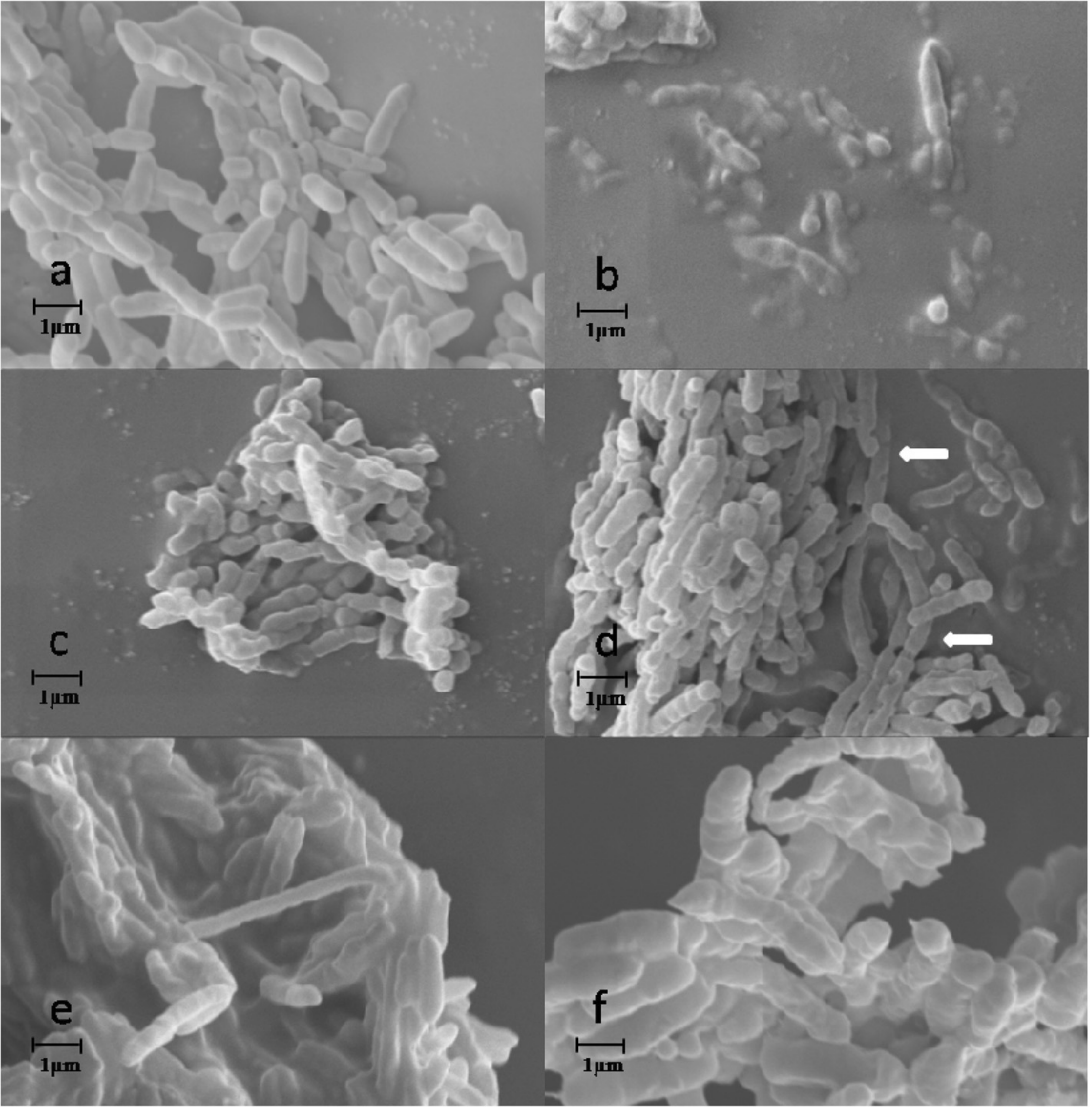
**SEM photographs of freeze-dried** ***P. fluorescens*** **cells cultured in the presence of 5% (v/v) ILs (a:water, b: [EMI][BF**_**4**_ **], c: [BMI][BF**_**4**_ **], d: [HMI][BF**_**4**_ **], e: [OMI][BF**_**4**_ **], f: [BMI][PF**_**6**_ **]), magnification was 10 K.** Arrows: partially dissolved cell.

A visual research of the morphology changes of *P. fluorescens* cells cultured with various ILs was also conducted by using SEM. Table 3 showed that the cell sizes of *P. fluorescens* was significantly affected by different IL-containing system. Figure 6a showed the SEM micrographs of *P. fluorescens* cells in their normal state, which are straight bacilliform with average size of 2.4 × 0.50 μm. Figure 6b-f shows the same cells in ILs-affected states. Compared with that in the control group, quite different and interesting morphology changes of *P. fluorescens* cells were found in terms of elongated cell sizes and rough surface with pits and bulges.

**Table 3:** **The cell size of** ***P. fluorescens*** **cells cultured in various medium**

	**IL added in culture media**					
**Cell size**	**Water**	**[EMI][BF**_**4**_ **]**	**[BMI][BF**_**4**_ **]**	**[HMI][BF**_**4**_ **]**	**[OMI][BF**_**4**_ **]**	**[BMI][PF**_**6**_ **]**
Length (μm)	2.4 ± 0.2	2.7 ± 0.7	2.0 ± 0.3	4.1 ± 1.7	3.1 ± 0.9	3.2 ± 0.9
Diameter (μm)	0.5 ± 0.1	0.5 ± 0.1	0.5 ± 0.1	0.5 ± 0.1	0.5 ± 0.1	0.7 ± 0.3

Rough surface with slight horizontal pits were found in cells cultured with the ILs containing the same anion but different cations with shorter alkyl carbon chain length (Figure 6b-d). When cultured in [BF_4_]-based ILs with increased alkyl carbon chains, the cells showed more pits and bulges spreading over the cell surface. And the cell wall was even dissolved partly as indicated by the arrows in Figure 6d. For [OMI][BF_4_], rough surfaces with slight horizontal pits of the cells were found (Figure 6e). Results further suggested that the cell wall, especially the outer layer of the bacterial cells may be involved in the toxicity effect of ILs on *P. fluorescens* cells, since the phospholipids bilayer of the out membrane may be damaged by the solvents [28]. And the most possible areas that the solvents acted on were the phospholipids outer membrane and plasma membrane of the Gram-negative bacteria tested. These influences caused by ILs during cultivation may thus effect the structural and functional integrity of the cell [34,35]. In the medium containing [BMI][PF_6_], larger cell size and more indistinct cell shape than those cultivated with other ILs were observed (Figure 6f). This result further confirmed that the hydrophobic IL [BMI][PF_6_] had higher effects on altering cell envelope, which may tend to accumulated at the surface of the cells in an aqueous culture medium and partially disrupt cell membranes. The results were in accordance with the surface changes of the cells harvested at exponential growth stage and treated with ILs. The results mentioned above confirmed that ILs may disrupt the cell membranes of *P. fluorescens*, which is harmful to living cells but useful for improving efficiency of whole-cell biocatalysis, since mass transfer limitation was especially serious in whole-cell biocatalysis [4].

## Conclusions

The research demonstrated the highly regioselective synthesis of long-chain nucleoside ester can be achieved by using *P. fluorescens* whole-cells in IL-organic systems. To our knowledge, this is the first report on the application of ILs in whole-cell catalyzed acylation of nucleosides. The influence of the ILs on the reaction closely depended on the types of both the anion and cation of the IL used. The good catalytic behaviors of whole-cells in the reaction medium with [BMI][PF_6_] may be due to its higher hydrophobicity and lower hydrogen bond basicity of the anion, as well as its capability of improving the permeability of cell envelope. This whole-cell strategy for bioactive nucleoside ester synthesis will be attractive for large-scale industrial application. The understanding of ILs influence on the cell morphology may be exploited to rationally acclimation and screening of IL-tolerant strains to achieve highly efficient biocatalysis.

## Methods

### Microorganisms and chemicals

*Pseudomonas fluorescens* GIM1.209 was supplied by the Guangdong Institute of Microbiology (GDIM), Guangzhou, China. Pure ara-C, 99+% VL and the ILs (97% [EMI][BF_4_], 97% [BMI][BF_4_], 97% [HMI][BF_4_], 98% [OMI][BF_4_], 97% [BMI][PF_6_], 97% [BMI][Cl], 97% [BMI][Br], 98% [BMI][TF_2_N]) were purchased from Sigma-Aldrich Chemical Co. (USA). All other chemicals were applied by Merk (Germany) and Tianjin Kemiou chemical reagent co., LTD, and were of the highest purity available.

### Whole-cell biocatalyst preparation

The precultivation was performed in the medium containing 1% glucose (all the percentage here referred to v/v), 1% beef extract, 1% peptone, 0.5% K_2_HPO_4_ and 0.5% NaCl at 30°C for 24 h. Then 2% seed culture was inoculated to the culture medium containing (g/L) (NH_4_)_2_SO_4_ 5.0, K_2_HPO_4_ 1.0, MgSO_4_**·**7H_2_O 0.2, soybean oil 5.5 and yeast extracts 1.0. To obtain whole-cell biocatalyst, the cultivation was carried out in 500 mL flasks containing 100 mL culture media on a rotary shaker at 30°C and 180 rpm. All the bacteria cells in 100 mL culture were harvested by centrifugation to remove the fluid medium, washed twice with distilled water, freeze-dried at -30°C for 24 h and then temporarily stored at 4°C for use in the subsequent reactions.

### General procedure for whole-cell catalyzed acylation of ara-C

In a typical reaction, 1 mL IL-containing solvents contained 20 mM ara-C, 900 mM VL, 4% water and 50 mg/mL freeze-dried cells, incubated by shaking (200 rpm) at 40°C for 144 hours. Samples for detection were taken at specified time intervals from the reaction mixture, and diluted 10×10 times with pure methanol prior to HPLC analysis. To structurally characterize the product of the reaction, the volume of components of the reaction was scaled up. Upon completion of the reaction, the reaction mixture was centrifuged to remove the cell masses, isolated, and purified by half-preparation HPLC. The acquisition was concentrated to about 1 mL by vacuum rotary evaporation. After crystallization under 4°C, two products were obtained as white powder. All reported data were averages of experiments performed at least in duplicate.

### Determination of Protein

The protein was detected with a Bradford Protein Assay KitSuper. After the finished reaction, the whole-cells was removed by syringe filters with 0.45 μm membrane and diluted with PBS to 1 mL, took 0.1 mL sample added to 1 mL Bradford Protein Assay Reagent,mixed sufficiently, reacted for 5 minutes and detected the optical density of 595 nm. All reported data were averages of experiments performed at least in duplicate.

### Thermal-stability assay

50 mg aliquots of dried cells were added into separate screw-capped vials containing 1 ml of the selected medium {10% (v/v) [BMI][BF_4_]-THF, 10% (v/v) [BMI][PF_6_]-THF or 80% (v/v) [OMI][BF_4_]-THF} and the mixture was incubated for 12 hours at various temperatures from 30 to 70°C. Then the biocatalyst was filtrated from the solvent mixtures and added to a fresh medium containing 20 mM ara-C and 900 mM VL to initial the reaction at 200 rpm under 40°C. The relative activity was expressed as the ratio of the residual catalytic activity of the biocatalyst after incubation at different temperatures to its original activity in the same reaction system.

### Bacteria growth in the presence of ILs

Bacteria growth in the presence of a second phase was determined based on a method described previously [36]. 2 mL seed culture of *P. fluorescens* after 24 h shaking the culture was transferred to new culture bottles (100 ml) and 5% (v/v) of ILs was added. The culture bottles were incubated at 30°C for 48 h. Finally the bacterial growth was evaluated by direct measurement of absorbance at 560 nm (OD_560_) using a pure culture fluid as blank control [37].

### SEM analysis of the cells treated with ILs or grown in IL-containing culture media

Bacterium were cultured in the media with the presence of 5% ILs, based on a method described previously [36]. And the freeze-dried cells were treated by incubation in different 10% (v/v) ILs-containing solvents at 30°C for 48 h (10% Pyridine was added to each solvent system as a solubilizer). After that, the bacterium mass was centrifuged, freeze-dried and sputter-coated with a thin layer of Au. Finally the coated cell samples were analyzed using a Zeiss EVO 18 scanning electron microscope (Germany) with 10 kV accelerating voltage in secondary electron mode. The magnification was 10 K in SEM micrographs.

### HPLC analysis

The reaction mixture was analyzed by RP-HPLC on a 4.6 mm × 250 mm (5 μm) Zorbax SB-C18 column (Agilent Technologies Co. Ltd, Massachusetts, USA) with Waters 600E pump and Waters 2996UV/photodiode array detector (Waters Corp., Massachusetts, USA) at 276 nm. A mixture of ammonium acetate buffer (0.01 M, pH 4.27) and methanol (88/12, v/v) was used as mobile phase at the flow rate of 0.9 mL/min. The retention times for ara-C, 3′-*O*-laurylara-C and 5′-*O*-lauryl ara-C were 2.79, 6.92 and 8.03 min, respectively. Regioselectivity was defined as the ratio of the indicated product’s HPLC peak area to that of all the products formed upon a certain reaction time.The5′-*O*-ester yield of the reaction was defined as [the moles of 5′-regioisomer in the acylation reaction] × 100%/(initial moles of ara-C). The average error for this determination was less than 1.0%.

### Structure determination of the products

The position of acylation in the biologically prepared ester was determined by ^13^C NMR (Bruker AVANCE Digital 400 MHz Nuclear Magnetic Resonance Spectrometer, Bruker Co., Germany) at 100 MHz. DMSO-*d*_6_ was used as solvent and chemical shifts were expressed in ppm shift. The LC-MS spectra of the product were recorded on an Agilent 1290/BrukerMaXis Impact Plus ESI Mass Spectrometer with a spray voltage of 4.5 kV (Bruker Co., Germany).

**Ara-C:**^13^C-NMR δ 164.9 (C-4), 154.3 (C-2), 143.4 (C-6), 92.5 (C-5), 86.5 (C-1′), 85.3 (C-4′), 76.3 (C-3′), 74.9 (C-2′), 61.2 (C-5′); FT-IR (KBr, cm-1) v 3214-3342 (OH,NH), 2922 (CH), 1649 (C = C), 1117/1051 (C-O-C).

**5′-O-lauryl ara-C**: ^13^C-NMR (DMSO-*d*_6_, 100 MH_Z_) δ: 173.29 (COO), 165.80 (C4-N), 155.62 (C = O 2), 143.37 (C-6), 93.35 (C-5), 86.97 (C-1′), 82.55 (C-4′), 77. 27 (C-3′), 75.09 (C-2′), 64.16 (C-5′), 22.52-34.04 (CH_2_), 13.81 (CH_3_). ESI-MS (C_21_H_35_N_3_O_6_, 425.53): m/z = 424.20(100) ([M-H]^+^).

**3′-O-lauryl ara-C**: ^13^C-NMR(DMSO-*d*_6_, 100 MH_Z_) δ: 172.36 (COO), 164.45 (C4-N), 155.85(C = O 2), 144.46 (C-6), 93.49 (C-5), 86.90 (C-1′), 83.02 (C-4′), 79. 16 (C-3′), 72.95 (C-2′), 61.42 (C-5′), 25.65-34.27 (CH_2_), 14.48 (CH_3_); ESI-MS (C_21_H_35_N_3_O_6_, 425.53): m/z = 424.20 ([M-H]^+^).

## Abbreviations

IL(s): Ionic liquid(s)
[EMI]^+^: 1-ethyl-3-methylimidazolium
[BMI]^+^: 1-butyl-3-methylimidazolium
[HMI]^+^: 1-hexyl-3-methylimidazolium
[OMI]^+^: 1-octyl-3-methylimidazolium
DMSO: Dimethylsulfoxide
VL: Vinyl laurate

## References

[1] Klibanov AM (2001). Improving enzymes by using them in organic solvents. Nature.

[2] de Carvalho CC (2011). Enzymatic and whole cell catalysis: Finding new strategies for old processes. Biotechnol Adv.

[3] Schmid A, Dordick J, Hauer B, Kiener A, Wubbolts M, Witholt B (2001). Industrial biocatalysis today and tomorrow. Nature.

[4] Duetz WA, Van Beilen JB, Witholt B (2001). Using proteins in their natural environment: potential and limitations of microbial whole-cell hydroxylations in applied biocatalysis. Curr Opin Biotechnol.

[5] Idris A, Bukhari A (2012). Immobilized *Candida antarctica* lipase B: Hydration, stripping off and application in ring opening polyester synthesis. Biotechnol Adv.

[6] Kalscheuer R, Stölting T, Steinbüchel A (2006). Microdiesel: Escherichia coli engineered for fuel production. Microbiology.

[7] Nóbile M, Médici R, Terreni M, Lewkowicz ES, Iribarren AM (2012). Use of Citrobacter koseri whole cells for the production of arabinonucleosides: A larger scale approach. Process Biochem.

[8] Ferrero M, Gotor V (2000). Biocatalytic selective modifications of conventional nucleosides, carbocyclic nucleosides, and C-nucleosides. Chem Rev.

[9] Li N, Smith TJ, Zong M-H (2010). Biocatalytic transformation of nucleoside derivatives. Biotechnol Adv.

[10] Li X-F, Lou W-Y, Smith TJ, Zong M-H, Wu H, Wang J-F (2006). Efficient regioselective acylation of 1-beta-D-arabinofuranosylcytosine catalyzed by lipase in ionic liquid containing systems. Green Chem.

[11] Ferrero M, Gotor V (2000). Chemoenzymatic transformationsin nucleoside chemistry. Monatsh Chem.

[12] Naushad M, Alothman ZA, Khan AB, Ali M (2012). Effect of ionic liquid on activity, stability, and structure of enzymes: A review. Int J Biol Macromol.

[13] Heipieper HJ, Neumann G, Cornelissen S, Meinhardt F (2007). Solvent-tolerant bacteria for biotransformations in two-phase fermentation systems. Appl Microbiol Biotechnol.

[14] Carrea G, Ottolina G, Riva S (1995). Role of solvents in the control of enzyme selectivity in organic media. Trends Biotechnol.

[15] Sheldon RA (2005). Green solvents for sustainable organic synthesis: state of the art. Green Chem.

[16] van Rantwijk F, Madeira Lau R, Sheldon RA (2003). Biocatalytic transformations in ionic liquids. Trends Biotechnol.

[17] Gathergood N, Scammells PJ (2002). Design and preparation of room-temperature ionic liquids containing biodegradable side chains. Aust J Chem.

[18] Kilpeläinen I, Xie H, King A, Granstrom M, Heikkinen S, Argyropoulos DS (2007). Dissolution of wood in ionic liquids. J Agric Food Chem.

[19] Li Q, He Y-C, Xian M, Jun G, Xu X, Yang J-M, Li L-Z (2009). Improving enzymatic hydrolysis of wheat straw using ionic liquid 1-ethyl-3-methyl imidazolium diethyl phosphate pretreatment. Bioresour Technol.

[20] Rahimi P, Ghourchian H, Sajjadi S (2012). Effect of hydrophilicity of room temperature ionic liquids on the electrochemical and electrocatalytic behaviour of choline oxidase. Analyst.

[21] Sieffert N, Wipff G (2006). The [BMI][Tf2N] ionic liquid/water binary system: A molecular dynamics study of phase separation and of the liquid-liquid interface. J Phys Chem B.

[22] Sheldon RA, Lau RM, Sorgedrager MJ, van Rantwijk F, Seddon KR (2002). Biocatalysis in ionic liquids. Green Chem.

[23] Lou W-Y, Zong M-H, Wu H, Xu R, Wang J-F (2005). Markedly improving lipase-mediated asymmetric ammonolysis of D, Lp-hydroxyphenylglycine methyl ester by using an ionic liquid as the reaction medium. Green Chem.

[24] Reichardt C (1979). Empirical Parameters of Solvent Polarity as Linear Free‐Energy Relationships. Angew Chem Int Ed Engl.

[25] Lozano P, De Diego T, Carrie D, Vaultier M, Iborra J (2001). Over-stabilization of Candida antarctica lipase B by ionic liquids in ester synthesis. Biotechnol Lett.

[26] Persson M, Bornscheuer UT (2003). Increased stability of an esterase from *Bacillus stearothermophilus* in ionic liquids as compared to organic solvents. J Mol Catal B Enzym.

[27] Cull S, Holbrey J, Vargas‐Mora V, Seddon K, Lye G (2000). Room‐temperature ionic liquids as replacements for organic solvents in multiphase bioprocess operations. Biotechnol Bioeng.

[28] Sena DW, Kulacki KJ, Chaloner DT, Lamberti GA (2010). The role of the cell wall in the toxicity of ionic liquids to the alga Chlamydomonas reinhardtii. Green Chem.

[29] Kieboom J, Dennis JJ, Zylstra GJ, de Bont JA (1998). Active Efflux of Organic Solvents byPseudomonas putida S12 Is Induced by Solvents. J Bacteriol.

[30] Nakajima H, Kobayashi K, Kobayashi M, Asako H, Aono R (1995). Overexpression of the robA gene increases organic solvent tolerance and multiple antibiotic and heavy metal ion resistance in Escherichia coli. Appl Environ Microbiol.

[31] Barahona D, Pfromm PH, Rezac ME (2006). Effect of water activity on the lipase catalyzed esterification of geraniol in ionic liquid bmim PF6. Biotechnol Bioeng.

[32] Ranke J, Cox M, Müller A, Schmidt C, Beyersmann D (2006). Sorption, cellular distribution, and cytotoxicity of imidazolium ionic liquids in mammalian cells–influence of lipophilicity. Toxicol Environ Chem.

[33] Łuczak J, Jungnickel C, Łącka I, Stolte S, Hupka J (2010). Antimicrobial and surface activity of 1-alkyl-3-methylimidazolium derivatives. Green Chem.

[34] Inoue A, Horikoshi K (1989). A Pseudomonas thrives in high concentrations of toluene. Nature.

[35] Sikkema J, De Bont J, Poolman B (1995). Mechanisms of membrane toxicity of hydrocarbons. Microbiol Rev.

[36] Matsumoto M, de Bont JA, Isken S (2002). Isolation and characterization of the solvent-tolerant *Bacillus cereus* strain R1. J Biosci Bioeng.

[37] Matsumoto M, Mochiduki K, Kondo K (2004). Toxicity of ionic liquids and organic solvents to lactic acid-producing bacteria. J Biosci Bioeng.

